# Harnessing albumin’s natural tumor-targeting properties: nanoplatform strategies for triple-negative breast cancer therapy

**DOI:** 10.1186/s11671-025-04411-7

**Published:** 2026-01-05

**Authors:** Mujibullah Sheikh, Deepak Khobragade, Aashita Sakore, Umesh Telrandhe

**Affiliations:** 1 Datta Meghe College of Pharmacy, Datta Meghe Institute of Higher Education and Research, (DU), Sawangi (M), Wardha, Maharashtra India; 2 Tulaskar College of Pharmacy, Hinganghat Dist. Wardha (M.S), 442301 India

**Keywords:** Albumin nanoparticles, TNBC, Targeted therapy, Nanomedicine, Drug delivery

## Abstract

Triple-negative breast cancer (TNBC) represents the most aggressive breast cancer subtype and is characterized by the absence of estrogen receptor, progesterone receptor, and HER2 expression; this subtype affects approximately 12–20% of all breast cancer cases, with a disproportionately poor prognosis and limited therapeutic options. The lack of targetable receptors excludes TNBC patients from hormone therapy and HER2-targeted treatments, resulting in the use of chemotherapy as the primary intervention, which is often associated with severe systemic toxicity and drug resistance. Albumin-based nanoplatforms have emerged as promising solutions to address these therapeutic challenges by exploiting the inherent biocompatibility, biodegradability, extended circulation half-life, and natural tumor-targeting properties of albumin through interactions with gp60 and SPARC receptors that are overexpressed in TNBC tissues. This comprehensive review examines the molecular design principles, fabrication strategies, and targeting mechanisms of albumin nanocarriers, including passive targeting via the enhanced permeability and retention (EPR) effect and active targeting through receptor‒ligand interactions with uPAR, EGFR, CD44, CXCR4, and folate receptors. We analyze diverse therapeutic payloads, including conventional chemotherapeutics (paclitaxel, doxorubicin, and docetaxel), natural products (curcumin and resveratrol), and molecular therapeutics (siRNAs and CRISPR/Cas9) delivered via albumin nanoplatforms. The clinical evidence supporting nab-paclitaxel in combination with immune checkpoint inhibitors has demonstrated significant improvements in progression-free survival and objective response rates in PD-L1-positive mTNBC patients, whereas real-world studies have confirmed manageable safety profiles. However, several challenges remain, including drug loading limitations, nanocarrier stability under physiological conditions, interpatient variability in EPR effectiveness, potential immunogenicity of modified albumin, and the inherent molecular heterogeneity of TNBC subtypes, which may require personalized approaches. Future directions emphasize the development of multistimuli-responsive albumin nanocarriers, integration with gene editing and immunotherapy, artificial intelligence-guided design optimization, and precision medicine strategies tailored to individual tumor profiles. The convergence of the natural tumor affinity of albumin with advanced nanotechnology holds substantial promise for overcoming drug resistance, enhancing therapeutic specificity, and improving clinical outcomes in TNBC patients, positioning albumin-based nanomedicine as a transformative approach in precision oncology.

**Graphical abstract:**

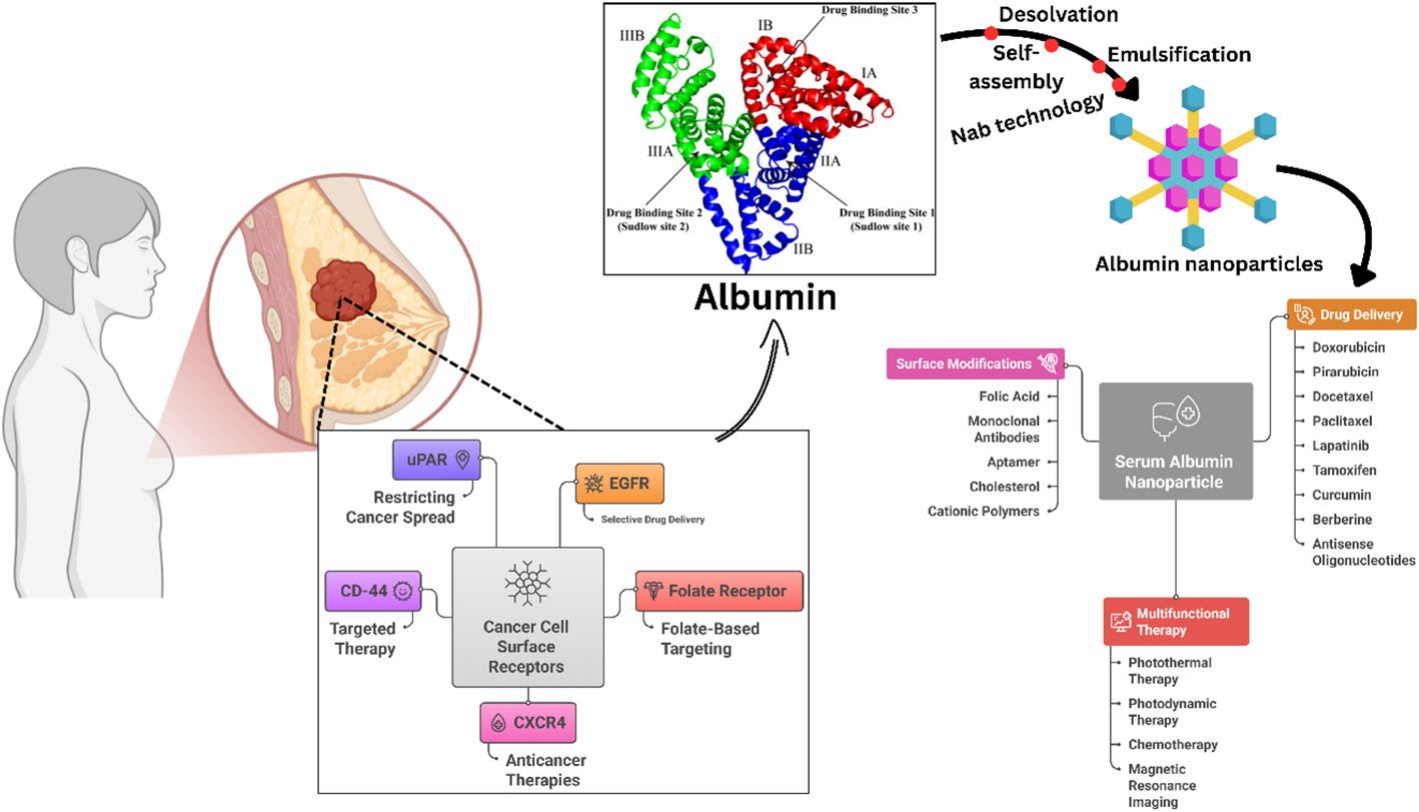

## Introduction

**Fig. 1:**
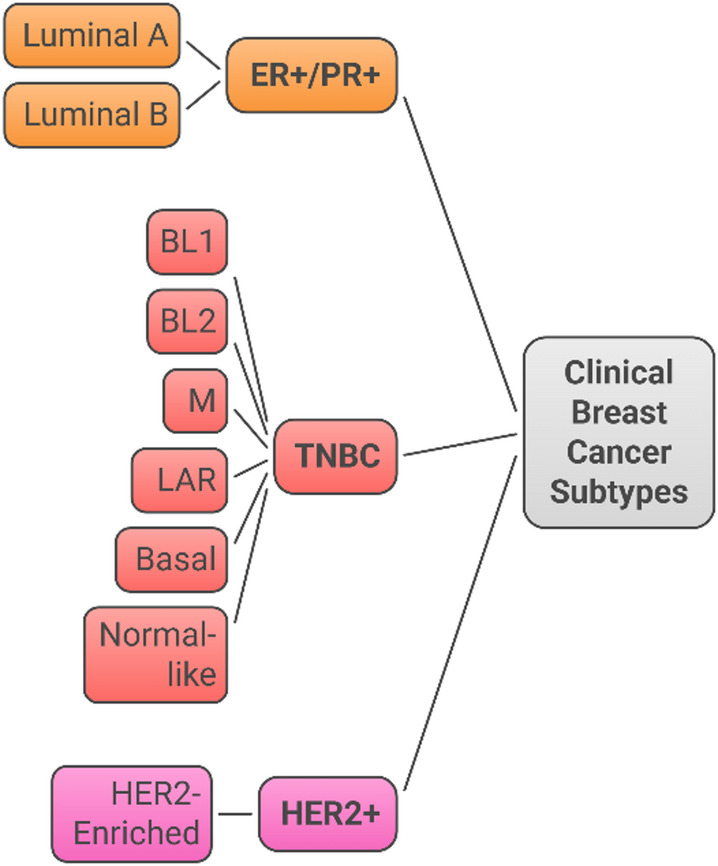
Schematic representation of the four primary clinical breast cancer subtypes based on hormone receptor and HER2 status. Estrogen receptor–positive/progesterone receptor–positive (ER + /PR +) tumors include luminal A and luminal B subtypes. HER2-enriched (HER2 +) tumors overexpress the HER2 oncogene. Triple-negative breast cancer (TNBC) lacks ER, PR, and HER2 expressions and comprises multiple molecular subtypes, including basal-like 1 (BL1), basal-like 2 (BL2), mesenchymal (M), luminal androgen receptor (LAR), basal, and normal-like variants

Breast cancer is a type of malignant tumor that is characterized by its clinical behavior, healing response and clinical manifestation (Fig. 1). The traditional classification system categorizes breast cancer into four subtypes on the basis of their hormone receptors (estrogen (ER), progesterone (PR), human epidermal growth factor receptor 2 (HER 2), and breast cancer (TNBC) [1, 2]. This molecular subclassification has significant implications for prognostic evaluation and therapy. Luminal A (ER + and/or PR + and HER2- low Ki-67) tumors usually have highly favorable outcomes and are responsive to endocrine therapy. Luminal B (ER + and/or PR + , HER2 + or high Ki-67) tumors are more virulent and may need multimodal treatment with the addition of chemotherapy and endocrine therapy [1]. Targeted anti-HER2 medicines are used in conjunction with standard treatments to treat HER2-enriched malignancies, which exhibit amplification of the HER2 oncogene [3].

The heterogeneity of breast cancer is therapeutic in origin and results from the molecular complexity that governs differential treatment responses. Gene expression profiling has further established breast cancer heterogeneity by defining several distinct biological classes with differential survival profiles [1]. Perou and coworkers initially showed via complementary DNA microarrays that breast tumors represent subtypes on the basis of their gene expression patterns, including luminal epithelial-like, basal-like, HER2-positive, and normal-like subgroups. Further studies have shown that these molecular subtypes correspond not only to distinct cells of origin but also to differentiated risk factors, metastatic patterns, and sensitivities to drugs, indicating the need for an individualized treatment approach [2].

Triple-negative breast cancer (TNBC), which is characterized by a lack of PR, ER and HER2 expression, accounts for approximately 12–20% of all breast cancers and is the most difficult type of cancer to manage. According to an epidemiological study, TNBC is unevenly distributed in women under 40 years of age, especially African American women who have hereditary BRCA1/2 mutations. Compared with other subtypes, the biological landscape of TNBC is characterized by aggressive tumor behavior, with a higher histological grade, increased proliferative index, and distinctive metastatic patterns. From a molecular perspective, TNBC encompasses several distinct entities, including basal-like 1 and 2, mesenchymal, and luminal androgen receptor subtypes, each with unique molecular features and therapeutic vulnerabilities (Table 1) [4, 5].

**Table 1:** Molecular characteristics, clinical features, genomic alterations, and therapeutic implications of key TNBC subtypes

Molecular subtype	Key gene expression characteristics	Genomic alterations	Clinical characteristics	Therapeutic implications	Frequency (%)	Prognosis
Basal-Like 1 (BL1)	Cell cycle and DNA damage response genes; Cell division pathways; BRCA1/BRCA2 dysfunction; High proliferation genes	TP53 mutations (high); BRCA1/2 mutations; RB1 loss; High genomic instability; DNA repair deficiency	Higher grade; Lower stage; Younger age at diagnosis; Better overall survival among TNBC; Higher pCR to neoadjuvant chemotherapy (41%)	DNA-damage repair inhibitors; Cell cycle modulators; PARP inhibitors; Platinum-based chemotherapy; High cisplatin sensitivity	20–25%	Intermediate to Good
Basal-Like 2 (BL2)	Growth factor signaling (EGFR, NGF, MET, Wnt/β-catenin); Myoepithelial markers; Glycolysis and gluconeogenesis pathways; E2F2 and TGFβ signaling	TP53 mutations; PIK3CA mutations; Growth factor receptor alterations; High genomic instability	Higher grade; Intermediate prognosis; Lower pCR rate (18%); May include metaplastic carcinomas	DNA alkylating agents; Growth factor receptor inhibitors; EGFR-targeted therapy; Standard chemotherapy	15–20%	Poor to Intermediate
Mesenchymal (M)	Epithelial–mesenchymal transition (EMT) genes; Cell motility and differentiation; Growth factor pathways; Angiogenesis signatures	TP53 mutations; EMT-related alterations; Angiogenesis pathway mutations; Stromal gene signatures	Intermediate prognosis; Preferential lung metastasis; Variable response to chemotherapy; Associated with angiogenesis	Anti-angiogenic agents; Kinase inhibitors; EMT pathway inhibitors; Stromal-targeting therapies	15–20%	Poor to Intermediate

The clinical challenges associated with TNBC are substantial. Patients with TNBC experience more frequent and early recurrences, particularly within the first 3–5 years after diagnosis, with a predilection for visceral metastases, including lung and brain involvement. The 5-year survival rate for TNBC patients is 8–16% lower than that for patients with hormone receptor-positive diseases, highlighting the urgent need for more effective therapeutic strategies. Notably, TNBC tumors represent only 8.2% of stage I cancers but constitute more than 15% of higher-stage diseases at diagnosis, indicating their aggressive nature and frequent late detection. Furthermore, the absence of established therapeutic targets excludes TNBC patients from receiving endocrine therapy or HER2-targeted agents, leaving conventional chemotherapy as the primary treatment option, to which many patients develop resistance [5, 6].

The current therapeutic arsenal for TNBC remains predominantly reliant on conventional modalities, including chemotherapy, radiotherapy, and, more recently, immunotherapy, all of which present significant limitations.

Chemotherapy, while serving as the cornerstone of TNBC treatment, has substantial shortcomings. Systemic chemotherapy agents (anthracyclines, taxanes, and platinum-based compounds) lack tumor-specific targeting ability, leading to widespread cytotoxic effects on rapidly dividing normal cells and consequent dose-limiting toxicities [7]. Common adverse effects include hematological suppression, neurotoxicity, nausea, vomiting, alopecia, and fatigue, which profoundly impact patients’ quality of life [8]. Moreover, chemotherapy often confers only modest benefits in the metastatic setting, with response rates diminishing upon disease recurrence owing to the emergence of multidrug resistance mechanisms [9]. Long-term complications may include irreversible organ damage (cardiotoxicity, nephrotoxicity), secondary malignancies, and premature menopause with associated infertility and bone health concerns [10].

Radiotherapy, which is frequently employed in the locoregional management of TNBC, presents several challenges. While effective in reducing local recurrence rates, radiotherapy can induce significant cosmetic changes in the treated breast, including fibrosis, telangiectasia, and tissue contraction [11, 12]. More concerning are the potential late effects, which may manifest years after treatment completion. These include cardiac toxicity (with a linear increase in major coronary events of 7.4% per 1 Gy of mean heart dose), pulmonary complications, and secondary malignancies such as lung cancer (with a rate ratio of 2.10 ≥ 10 years after radiation). The reconstructed breast poses additional challenges for radiotherapy planning and delivery, with increased rates of capsular contracture and compromised cosmetic outcomes [11].

Immunotherapy, particularly immune checkpoint inhibitors (ICIs), has emerged as a promising strategy for TNBC treatment. However, its efficacy remains limited to a subset of patients. ICIs such as atezolizumab and pembrolizumab demonstrate clinical activity primarily in PD-L1-positive TNBC, which represents only approximately 41% of the population [13]. Even in responsive patients, the absolute median survival benefit remains modest (approximately 2.5 months in the IMpassion130 trial). Significant challenges include primary and acquired resistance mechanisms, immune-related adverse events, the immunosuppressive tumor microenvironment, and the lack of reliable predictive biomarkers beyond PD-L1 expression. Additionally, the combination of ICIs with chemotherapy necessitates careful consideration of steroid premedication, which may dampen the immune response [3, 13].

Nanotechnology has emerged as a transformative approach to overcome the limitations of conventional cancer therapeutics. By exploiting the unique physicochemical properties of materials at the nanoscale (typically 1–100 nm), nanocarriers can increase the solubility and stability of therapeutic agents, prolong their circulation half-life, and improve their biodistribution to target sites [14]. The enhanced permeability and retention (EPR) effect, a phenomenon whereby nanoscale particles preferentially accumulate in tumor tissues due to their leaky vasculature and impaired lymphatic drainage, provides a foundation for the passive targeting of nanotherapeutics [15].

Numerous nanoplatforms, including liposomes, polymeric nanoparticles, dendrimers, inorganic nanoparticles, and protein-based nanocarriers, have been developed for cancer drug delivery. Each platform offers distinct advantages and limitations concerning loading capacity, stability, biocompatibility, and functionalization potential. Liposomal formulations (e.g., pegylated liposomal doxorubicin) and antibody‒drug conjugates (e.g., ado-trastuzumab emtansine, sacituzumab govitecan) represent some of the most clinically advanced nanotechnology-based approaches in breast cancer treatment [16, 17]. These agents have the ability to improve the therapeutic index of conventional chemotherapeutics by enhancing antitumor efficacy while simultaneously reducing systemic exposure and associated toxicities [18].

The application of nanotechnology in TNBC treatment holds particular promise given the urgent need for more effective and targeted therapeutic strategies. Nanocarriers can be engineered to simultaneously deliver multiple therapeutic agents (e.g., chemotherapeutic drugs with immunomodulators) to address tumor heterogeneity and overcome resistance mechanisms [19]. Furthermore, surface functionalization with targeting ligands (e.g., antibodies, peptides, and aptamers) can actively direct nanotherapeutics to tumor cells expressing specific surface markers, enhancing specificity and reducing off-target effects [20].

Among the various protein-based nanocarriers, albumin has emerged as a particularly promising platform for cancer drug delivery, especially in the context of TNBC. As a natural transport protein in human plasma, albumin offers several unique advantages as a drug delivery vehicle. Its inherent biocompatibility and biodegradability minimize the risk of adverse immune reactions and long-term accumulation concerns associated with synthetic carriers [21]. The naturally extended half-life (approximately 19 days in humans) of albumin results from its size (66.5 kDa) and interaction with the neonatal Fc receptor (FcRn), which protects it from catabolism and facilitates recycling, thereby promoting prolonged circulation of albumin-bound therapeutics [22, 23].

The abundant binding sites on albumin allow for the noncovalent conjugation of a wide range of therapeutic agents, including hydrophobic chemotherapeutics (e.g., paclitaxel and docetaxel) and immunomodulatory compounds [21]. This binding capacity enables the efficient delivery of poorly water-soluble drugs without requiring harsh solubilizing excipients that often contribute to treatment-related toxicity (e.g., Cremophor EL in conventional paclitaxel formulations) [24]. The clinical validation of albumin as an effective drug carrier is exemplified by the success of nab-paclitaxel (albumin-bound paclitaxel, Abraxane®), which has demonstrated superior efficacy and reduced toxicity compared with solvent-based paclitaxel in multiple cancer types, including TNBC [25].

In addition to its role as a passive carrier, albumin appears to participate in active targeting mechanisms in TNBC. Many aggressive tumors, including TNBC, exhibit increased metabolism and nutrient demand, leading to increased albumin uptake via receptor-mediated pathways such as gp60 (albumin-binding glycoprotein) and SPARC (Secreted Protein Acidic and Rich in Cysteine) [26]. SPARC is particularly overexpressed in the tumor microenvironment of TNBC, potentially facilitating the accumulation of albumin-bound therapeutics within both tumor cells and stromal components [27].

The versatility of albumin as a platform enables the development of multifunctional nanocarriers that can simultaneously deliver chemotherapeutic agents, immune modulators, and diagnostic contrast agents, facilitating theranostic applications. Furthermore, albumin nanoparticles can be surface modified with targeting ligands to increase their specificity for TNBC cells expressing specific surface markers (e.g., EGFR, CD44, or folate receptors) [28]. These unique properties position albumin-based nanoplatforms as particularly promising vehicles for addressing the numerous therapeutic challenges in TNBC, potentially overcoming drug resistance, reducing systemic toxicity, and improving overall treatment outcomes.

Albumin-based delivery systems have already achieved significant clinical success, establishing a strong foundation for their continued development in oncology. The FDA-approved formulation Abraxane® (nanoparticle albumin-bound paclitaxel) exemplifies this approach, utilizing albumin to solubilize paclitaxel without toxic solvents such as Cremophor EL, thereby improving tolerability, enabling higher dose intensity, and reducing infusion times [29–31]. Mechanistically, Abraxane® leverages albumin’s interaction with gp60 and SPARC receptors to facilitate receptor-mediated transcytosis, enhance tumor accumulation, and increase intracellular drug delivery [32–34]. Similarly, Fyarro® (nab-sirolimus), the first albumin-bound mTOR inhibitor approved by the FDA, improves the solubility and bioavailability of sirolimus while maintaining a favorable safety profile [30, 35]. Table 2 summarizes key FDA-approved and investigational albumin nanoparticle chemotherapeutic formulations, including Abraxane®, Fyarro®, and next-generation candidates such as ABI-008 (nab-docetaxel) and ABI-011 (nab-thiocolchicine dimer), which are undergoing phase I/II clinical trials [35, 36]. These examples illustrate how albumin’s inherent biocompatibility, receptor-mediated transport, and long circulatory half-life have been successfully translated from preclinical validation to clinical benefit. Despite these advances, current nab technologies still face challenges such as limited compatibility with hydrophilic drugs, high production costs, and dependence on proprietary manufacturing platforms. Addressing these limitations remains central to developing the next generation of multifunctional albumin nanocarriers for targeted triple-negative breast cancer (TNBC) therapy [30].

**Table 2:** FDA-approved and investigational albumin-based nanoparticle chemotherapeutic formulations for triple-negative breast cancer, showing regulatory status, mechanisms, and key clinical benefits

Product	Active agent	Indication	FDA approval date	Clinical trial number	Mechanism highlights	Key benefits
Abraxane	Nab-paclitaxel	Metastatic breast cancer	January 2005	Phase III (multiple)	Albumin nanoparticle formulation avoiding Cremophor EL	Increased intracellular delivery; higher dose intensity; shorter infusion time
Abraxane	Nab-paclitaxel	PD-L1-positive unresectable locally advanced or metastatic TNBC (with atezolizumab)	March 2019	NCT02425891 (IMpassion130)	Albumin receptor-mediated endocytosis; SPARC-binding; enhanced tumor uptake	Enhanced tumor uptake; higher tumor-to-plasma ratio; no premedication required
Fyarro	Nab-sirolimus	Advanced malignant PEComa	November 2021	Phase II trials	Albumin-bound mTOR inhibitor	Improved solubility and bioavailability of sirolimus
In Development						
ABI-008	Nab-docetaxel	Hormone-refractory prostate cancer (Phase I/II)	Not approved	Phase I/II trials	Albumin-bound docetaxel formulation	Potential for reduced toxicity compared to conventional docetaxel
ABI-011	Nab-thiocolchicine dimer	Various solid tumors (Phase I)	Not approved	Phase I trials	Albumin-bound microtubule inhibitor	Enhanced delivery to tumor sites
Clinical Trials						
Abraxane	Nab-paclitaxel	Neoadjuvant TNBC	-	NCT01822314 (ETNA)	Weekly nab-paclitaxel vs paclitaxel before anthracyclines	Numerically higher pCR rates in TNBC subgroup
Abraxane	Nab-paclitaxel	Metastatic TNBC with tigatuzumab	-	NCT01307891 (TBCRC 019)	Combination with anti-DR5 antibody	Enhanced apoptosis induction; prolonged PFS in subset of patients
Abraxane	Nab-paclitaxel	Visceral metastases breast cancer	-	NCT02687490	Weekly dosing for high-risk metastatic disease	Median PFS 5.1 months; acceptable safety profile in Asian patients
Tinengotinib + Abraxane	Tinengotinib + nab-paclitaxel	Advanced TNBC combination therapy	-	NCT04742959	Multikinase inhibitor with albumin-bound paclitaxel	Promising clinical signals; well-tolerated combination

This review comprehensively examines the current landscape of albumin-based nanoplatforms for TNBC therapy and explores their design principles, mechanisms of action, and preclinical and clinical evidence supporting their application. We discuss how these innovative approaches address the limitations of conventional therapies and explore future directions for harnessing the full potential of albumin nanotechnology in the precision medicine era for TNBC.

## Albumin: structure, properties, and clinical relevance

### Molecular structure of human serum albumin

**Fig. 2:**
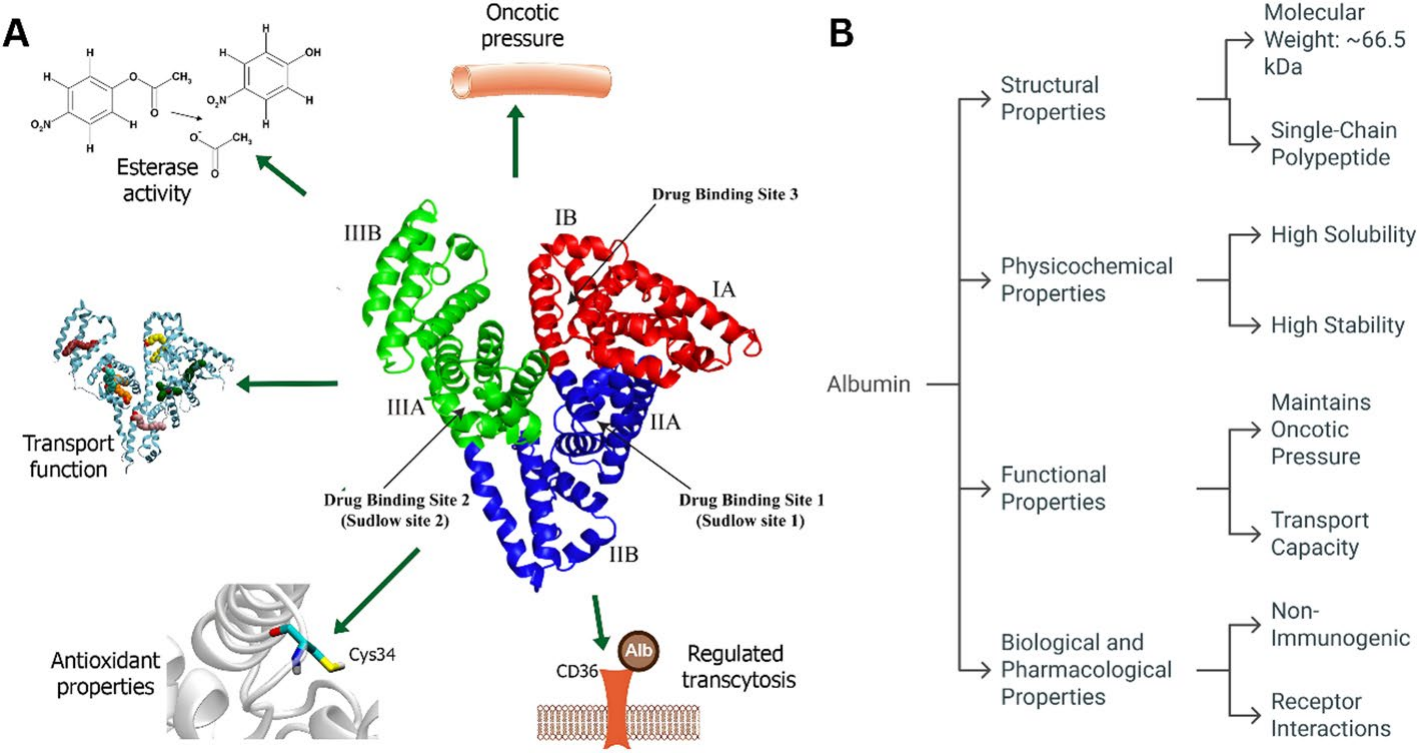
Structural and functional overview of HSA. (A) Representation of albumin’s structural domains and drug binding sites (Sudlow site 1, Sudlow site 2, and drug binding site 3) along with its diverse biological roles, including esterase activity, antioxidant properties via Cys34, transport function, maintenance of oncotic pressure, and regulation of transcytosis through CD36-mediated pathways. (B) Key properties of albumin categorized into structural properties (molecular weight and single-chain polypeptide), physicochemical properties (high solubility and stability), functional properties (maintenance of oncotic pressure and transport capacity), and biological/pharmacological properties (nonimmunogenic nature and receptor interactions)

HSA is a globular-shaped protein composed of a single 585 amino acid polypeptide and has a molecular weight of 66.5 kDa [37, 38]. It has a secondary structure that consists of 67% alpha helices, 10% beta turns and 23% extended chains. Structurally, HSA is divided into three homologous domains (I, II, and III), each further split into two subdomains (A and B) that fold to create the typical heart-shaped conformation [39]. The stability of the protein is ensured by 17 disulfide bridges created by 34 cysteine residues, one of which is a single free cysteine residue (Cys34) found in a hydrophobic crevice that is important for the antioxidant character of the protein [40]. The primary drug-binding sites are located in hydrophobic cavities within subdomains IIA and IIIA, designated Sudlow’s Site I and Site II, respectively. Sudlow Site I, positioned in subdomain IIA, preferentially binds bulky heterocyclic compounds such as warfarin, phenylbutazone, and azapropazone [37], whereas Sudlow Site II in subdomain IIIA has a high affinity for aromatic compounds such as ibuprofen, diazepam, and flufenamic acid. In addition to these primary binding sites, HSA possesses multiple secondary binding sites distributed across all three domains, including at least seven fatty acid binding sites, with sites 2, 4, and 5 exhibiting high affinity and sites 1, 3, 6, and 7 showing lower affinity. This extensive binding capacity, combined with HSA’s long circulatory half-life of 16–18 h and conformational flexibility, enables the protein to serve as the principal transport vehicle for a remarkably wide range of endogenous compounds and pharmaceutical agents in human plasma [41]. The multidomain structure and multifunctional properties of HAS, including drug binding, antioxidant activity, esterase activity, transport, and maintenance of oncotic pressure, are illustrated in Fig. 2.

Given the remarkable structural complexity and multifunctional nature of HSA, its biocompatibility and biodegradability profile make it an exemplary therapeutic protein with an outstanding clinical safety record. The inherent biocompatibility of albumin stems from its endogenous origin, absence of heterologous surface groups, and hydrophilic protein surface characteristics that preclude immune reactions and ensure excellent tolerance in biological systems. Clinical safety evidence spanning over eight decades demonstrates the exceptional safety profile of HSA, with the largest meta-analysis encompassing 26,351 patients across 58 clinical trials revealing no significant difference in mortality rates compared with alternative volume expanders. In critically ill patients, large-scale studies, including the SAFE trial with 6,997 participants and the Cochrane analysis of 10,842 patients, have consistently demonstrated the safety equivalence of albumin to crystalloid solutions, with no detectable signal of harm. The protein undergoes controlled enzymatic degradation through proteolytic pathways, which are primarily mediated by cellular proteases in lysosomes and secretory granules, resulting in the generation of bioactive peptides that may possess additional physiological functions. HSA has a remarkably long plasma half-life of approximately 19–21 days in humans and is regulated by the neonatal Fc receptor (FcRn), which protects the protein from intracellular degradation through pH-dependent binding and recycling mechanisms. However, enzymatic cleavage at the C-terminal leucine residue (L585) by carboxypeptidases can significantly reduce this half-life to 3.5 days, although engineered albumin variants can be designed to maintain optimal FcRn binding despite potential C-terminal modifications. The biodegradability of albumin-based formulations has been confirmed through in vivo studies demonstrating controlled degradation rates of 23–53% over 28 days, with degradation kinetics that can be modulated through formulation parameters. This combination of exceptional biocompatibility, predictable biodegradation, and extensive clinical safety validation positions human serum albumin as the gold standard carrier protein for pharmaceutical applications, with adverse events being exceptionally rare and primarily limited to mild reactions such as transient hypotension or localized inflammatory responses at therapeutic doses.

Human serum albumin (HSA) has unique pharmacokinetic properties, primarily driven by its interaction with the neonatal Fc receptor (FcRn). After being taken up into acidic endosomal compartments, FcRn binds to albumin and rescues it from lysosomal degradation by recycling it back to the cell surface. This FcRn-mediated recycling process significantly extends the half-life of HSA in circulation to approximately three weeks, facilitating its stable presence in plasma and enabling prolonged delivery of albumin-bound drugs. The extended circulation time also augments passive tumor accumulation through the enhanced permeability and retention (EPR) effect, where the leaky vasculature and poor lymphatic drainage of tumors allow albumin to preferentially extravasate and persist within tumor tissues.

In addition to being passively accumulated, albumin displays natural tumor affinity via interactions with specific receptors, such as glycoprotein 60 (gp60), on vascular endothelial cells and secreted protein acidic and rich in cysteine (SPARC), which is often overexpressed in tumor cells. Gp60 facilitates transcytosis of albumin across the endothelium, while SPARC binds and retains albumin within the tumor microenvironment, increasing the specificity of its cellular uptake and drug delivery. This receptor-mediated internalization ensures the selective delivery of albumin-bound therapeutics into cancer cells beyond what is afforded by the EPR effect alone. Together, these mechanisms underpin the capacity of albumin as a natural carrier that preferentially accumulates and releases therapeutics in tumors, improving its efficacy and minimizing systemic toxicity.

These advantageous properties have been harnessed in FDA-approved drug formulations such as the nanoparticle albumin-bound paclitaxel (nab-paclitaxel), which is used to treat metastatic breast, lung, and pancreatic cancers. Nab-paclitaxel exploits albumin’s solubilizing capacity and tumor-targeting mechanisms to enhance the delivery of paclitaxel, achieving higher tumor drug concentrations and improved therapeutic responses while reducing the solvent-related toxicity common to conventional formulations. In addition to nab-paclitaxel, many albumin-based formulations and nanoparticles are under development, leveraging the safety, biocompatibility, and inherent tumor-homing capabilities of albumin to create more effective and targeted cancer therapies. This convergence of pharmacokinetics, natural tumor affinity, and clinical application underscores the pivotal role of albumin in advancing drug delivery and oncology treatment paradigms.

## Strategies for albumin nanoparticle fabrication

The desolvation method, often termed coacervation, forms the backbone of many albumin nanoparticle preparations; it involves the controlled dropwise addition of a desolvating agent (ethanol, acetone, or methanol) into an aqueous albumin solution under constant stirring until turbidity increases nanoparticle formation, followed by stabilization through chemical crosslinking with agents such as glutaraldehyde or carbodiimide (EDC) to harden inter- and intramolecular amino‒to‒amino bonds and prevent redissolution or aggregation [42]. In self-assembly approaches, albumin‒drug conjugates exploit intrinsic hydrophobic domains: partial reduction of disulfide bonds (e.g., via dithiothreitol or glutathione) or conjugation to hydrophobic moieties exposes nonpolar pockets, enabling noncovalent loading of lipophilic drugs and spontaneous nanoparticle formation under mild heating or ionic strength conditions without crosslinkers, thereby preserving protein secondary structure while achieving high drug-loading efficiencies [43, 44]. The emulsification method encapsulates albumin within oil-in-water or water-in-oil emulsions: albumin is dispersed in the aqueous phase and emulsified with an immiscible organic solvent containing the drug, followed by solvent removal and nanoparticle solidification; this technique allows tunable particle sizes but often requires surfactants and extensive solvent removal to ensure biocompatibility [45]. Nab-technology (nanoparticle albumin-bound) refines emulsification by leveraging high-pressure homogenization of a paclitaxel-solvent phase into a presaturated albumin solution, yielding ~ 130 nm colloidal suspensions without crosslinkers; subsequent ultrafiltration removes residual solvents, producing reproducible, surfactant-free nanoparticles exemplified by FDA-approved nab-paclitaxel (Abraxane) with ~ 10% drug loading [45, 46]. Comparatively, desolvation is highly scalable and reproducible with minimal equipment needs but relies on toxic crosslinkers; self-assembly offers biocompatibility and high drug loading but faces scale-up challenges; emulsification enables versatile drug encapsulation but demands rigorous solvent control; and nab technology combines clinical-grade reproducibility and stability with moderate drug loading, positioning it as the current gold standard for albumin nanoparticle fabrication [42, 47]. Table 3 compares major albumin nanoparticle fabrication techniques in terms of scalability, reproducibility, stability, advantages, and limitations. Desolvation provides high reproducibility but uses toxic crosslinkers, self-assembly leverages mild conditions yet lacks scalability, emulsification offers versatility but demands surfactant optimization, and nab-technology achieves the best clinical applicability, although at higher cost.

**Table 3:** Comparison of albumin nanoparticle fabrication methods based on scalability, reproducibility, stability, advantages, and limitations

Fabrication method	Scalability	Reproducibility	Stability	Key advantages	Main limitations	Ref
Desolvation/Coacervation	Moderate to High	High (with process optimization and automation)	High (with cross-linking)	Simple setup; high drug loading capacity; controllable particle size; applicable to both HSA and BSA	Residual toxic cross-linkers (e.g., glutaraldehyde); requires purification steps; pH and flow rate sensitivity	[42, 47, 48]
Emulsification	Moderate	Moderate (requires surfactant optimization)	Moderate (dependent on stabilizers)	Suitable for hydrophobic drugs; uses mild conditions; avoids organic solvents if IL-based	Broad size distribution; potential surfactant toxicity; lower encapsulation efficiency for hydrophilic drugs	[49, 50]
Thermal Gelation	High	High (temperature-controlled)	High (thermally stable)	Solvent-free; no cross-linkers needed; simple and cost-effective; high biocompatibility	Limited to thermostable drugs; potential protein denaturation; less control over particle size	[42, 51]
Nab Technology	High (commercially proven)	High (standardized process)	Very high (lyophilizable)	FDA-approved (Abraxane); avoids solvents; high tumor targeting via SPARC and gp60 receptors; scalable production	Proprietary technology; high cost; limited to hydrophobic drugs	[47, 49, 51]
Self-Assembly	Low to Moderate	Moderate (dependent on drug-protein interactions)	Moderate (pH and ionic strength dependent)	Mild conditions; no external cross-linkers; high drug loading for specific ligands	Limited to drugs with high albumin affinity; variable batch-to-batch consistency; difficult to control size	[42, 51]
Nanospray Drying	High	High (process automated)	High (dry powder form)	Continuous production; narrow size distribution; suitable for thermolabile drugs; scalable	High equipment cost; potential nozzle clogging; optimization required for each formulation	[42]
Water-in-Ionic Liquid Microemulsion	Low (emerging method)	Moderate (requires IL purity control)	High (ILs enhance stability)	Green solvent alternative; tunable droplet size; high encapsulation efficiency; low toxicity	Cost of ionic liquids; complex phase behavior; limited long-term toxicity data	[50]

## Mechanisms by which albumin nanoplatforms target TNBC

### Passive targeting via the enhanced permeability and retention (EPR) effect

Passive targeting via the EPR effect leverages the unique pathophysiological features of solid tumors, namely, aberrant angiogenesis resulting in discontinuous endothelial linings with fenestrations of 100–800 nm and dysfunctional pericyte coverage, which allow macromolecules and nanoparticles to extravasate selectively into the tumor interstitium. Once within the tumor microenvironment, impaired lymphatic drainage fails to clear these agents efficiently, leading to prolonged retention and sustained therapeutic concentrations of these agents compared with those in normal tissues. Nanoparticle accumulation via the EPR effect is highly dependent on physicochemical parameters: optimal size (10–100 nm), neutral to slight negative surface charge, and hydrophilic coatings (e.g., polyethylene glycol) increase the circulation half-life and tumor uptake. Molecular mediators such as nitric oxide, bradykinin, vascular endothelial growth factor, and inflammatory cytokines further increase vascular permeability by loosening endothelial tight junctions and promoting transcytosis pathways. Pharmacological modulation strategies, including the administration of angiotensin II, nitroglycerin, and bradykinin analogs, have led to up to 3- to fourfold increases in EPR-mediated nanoparticle delivery in preclinical tumor models. Preclinical studies have reported that EPR-based delivery systems can achieve 10- to 20-fold higher intratumoral drug concentrations and improved antitumor efficacy than their free drug counterparts can achieve, highlighting the importance of passive targeting in nanomedicine design.

**Fig. 3:**
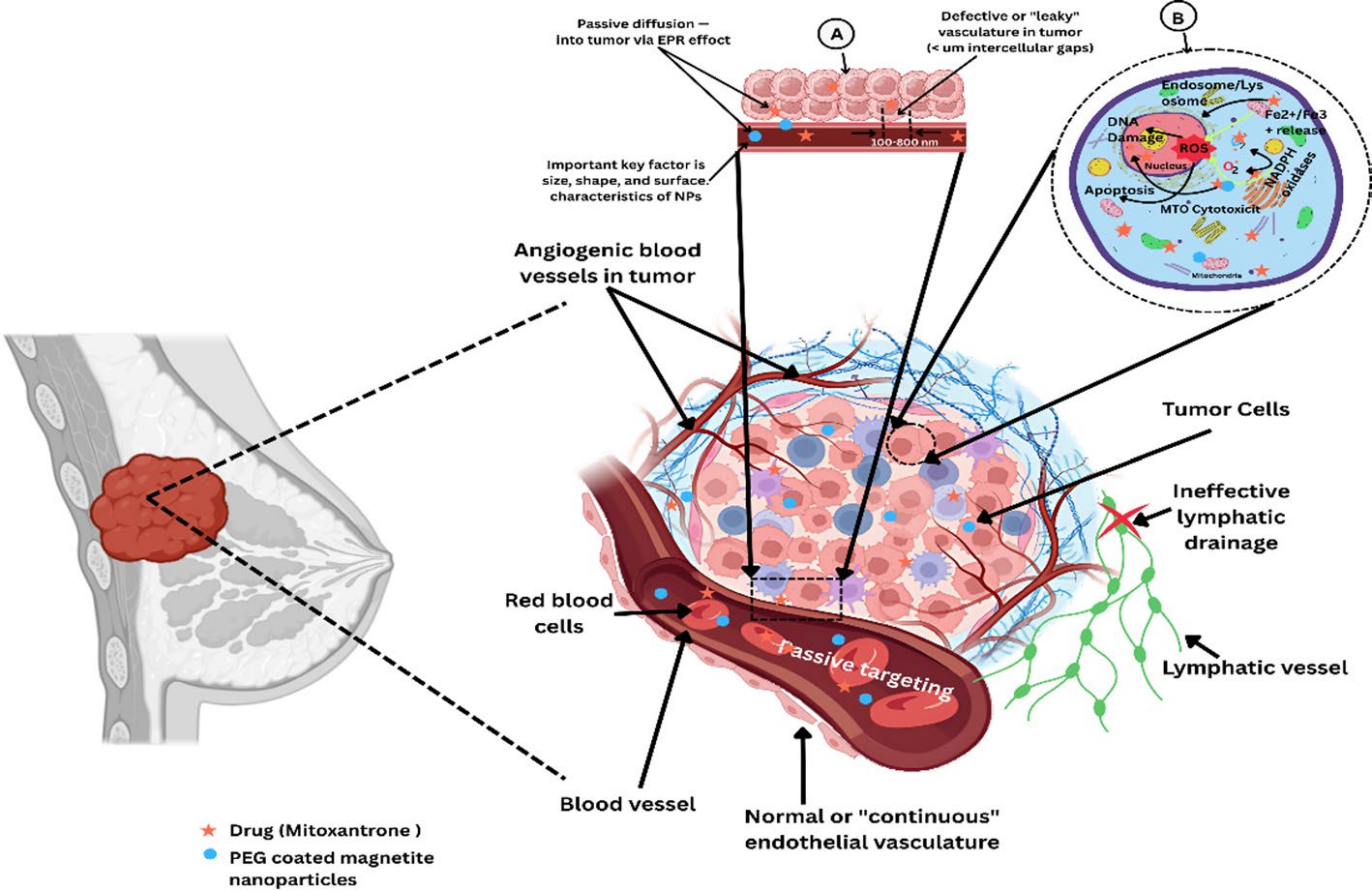
Schematic illustration of the passive targeting of PEG-coated magnetite nanoparticles and mitoxantrone in breast cancer via the EPR effect. The diagram depicts the accumulation of nanoparticles and drugs within angiogenic tumor blood vessels due to leaky vasculature and ineffective lymphatic drainage, facilitating their passive diffusion and retention in the tumor microenvironment. Insets highlight vascular endothelial characteristics and nanoparticle-induced cellular responses, including ROS generation, DNA damage, and apoptosis within tumor cells

However, inter- and intratumoral heterogeneity in vascular density, permeability, and extracellular matrix composition leads to variable EPR efficacy across tumor types and individual patients, posing challenges for clinical translation. Tumors such as breast, ovarian, and pancreatic cancers often exhibit more pronounced EPR effects due to higher microvessel density and increased protease activity, degrading the extracellular matrix, whereas tumors with dense stroma, such as pancreatic ductal adenocarcinoma, display limited nanoparticle penetration despite vascular leakiness. To visualize this phenomenon, the accompanying figure illustrates that PEG-coated magnetite nanoparticles and mitoxantrone start penetrating through the leaky tumor vasculature and accumulating within the tumor mass, underscoring the core principles of passive targeting via the EPR effect (Fig. 3).

### Active targeting through receptor‒ligand interactions

Active targeting through receptor‒ligand interactions in albumin-based nanoplatforms offers a sophisticated and highly effective approach for the targeted delivery of anticancer agents in triple-negative breast cancer (TNBC) [52]. Albumin, a naturally abundant protein in the human body, possesses intrinsic properties that make it an excellent carrier for drug delivery, including biocompatibility, biodegradability, and the ability to bind to various drugs and targeting ligands. In the context of TNBC, which lacks hormone receptor and HER2 expression, conventional therapies often face limitations due to the aggressive nature and heterogeneity of the disease [53, 54]. Albumin nanoparticles can be functionalized on their surface with specific ligands such as peptides (e.g., cRGDyK), antibodies, or small molecules that have high affinity for receptors overexpressed on TNBC cells or their microenvironment. These ligands guide the nanoparticles to bind specifically to receptors such as integrins (αvβ3), SPARC (secreted protein acidic and rich in cysteine), and other tumor-associated markers, facilitating receptor-mediated endocytosis [47]. Such active targeting strengthens the accumulation of the albumin nanoplatforms in tumor tissue over the passive EPR-enhanced effect, increasing the drug concentration in the tumor while reducing systemic toxicity. Albumin interacts with cellular receptors such as gp60, and penetration occurs through endothelial cells with the help of transcytosis, which promotes deep tumor diffusion [55]. Multifunctional albumin-based nanoparticles are formed that deliver chemotherapeutic drugs, photosensitizers, or genetic material. By combining chemotherapy with targeted gene therapy and photodynamic therapy, synergistic activity can be achieved. These nanoplatforms demonstrate improved pharmacokinetics, better tumor specificity, and fewer side effects, showing promising potential to overcome the challenges associated with TNBC treatment by enhancing therapeutic efficacy via controlled and stimuli-responsive drug release mechanisms. This strategy exemplifies a paradigm shift to precision nanomedicine in treating aggressive cancers such as TNBC by harnessing receptor‒ligand active targeting through albumin-based nanoplatforms [56].

#### uPAR

uPAR-directed albumin-based delivery leverages two complementary biological facts to improve drug targeting in breast cancer: uPAR is frequently overexpressed on invasive, chemo-resistant, and cancer stem-like breast tumor cells, driving invasion and Wnt/β-catenin signaling, and albumin is a long-circulating, easily modified carrier that cancer cells avidly scavenge via receptor-mediated routes and macropinocytosis. By decorating albumin or albumin-like carriers with uPA-derived peptides or ATF fragments that bind uPAR, nanoparticles gain receptor-mediated uptake and intracellular internalization into the very cell subpopulations that fuel recurrence, as shown by dual uPAR/Wnt-targeted iron oxide nanoparticles which increased tumor delivery, downregulated CD44 and uPAR, and improved antitumor efficacy in chemo-resistant breast PDX models [57]. Combining uPAR targeting with albumin chemistry also exploits albumin’s natural transcytosis and tumor accumulation pathways, including gp60/CAV1, SPARC interactions in stroma, and FcRn-dependent recycling, which together increase tumor exposure and can extend systemic half-life, while albumin modifications such as redox-responsive or ligand-conjugated derivatives enable triggered intracellular release and CD44 co-targeting when appropriate [58]. In practice this strategy can take several forms, for example albumin nanoparticles or albumin-coated nanocarriers carrying chemotherapeutics or oligonucleotides, and peptide or ligand conjugation improves selective uptake into uPAR-high niches, reduces off-target exposure, and can help overcome efflux-based resistance by delivering payloads directly into endosomal and cytosolic compartments [59]. Notably, recent work using uPA-targeted dendrimer gel nanoparticles to deliver the antisense oligonucleotide GTI-2040 against ribonucleotide reductase R2 in triple-negative breast cancer exemplifies how uPA/uPAR targeting combined with engineered carrier chemistry produces marked increases in cellular delivery, robust target knockdown, and significant tumor growth inhibition, indicating a practical path to translate uPAR-albumin design principles into effective therapies [60].

#### EGFR

Epidermal growth factor receptor (EGFR) and albumin-based systems have emerged as key platforms for targeted and efficient drug delivery in breast cancer therapy, particularly for TNBC, which lacks classical hormonal and HER2 targets. EGFR is frequently overexpressed in TNBC cells, making it an ideal molecular target for site-specific delivery of chemotherapeutics or gene therapeutics [61–63]. Anti-EGFR strategies employ a range of ligands, including monoclonal antibodies such as cetuximab, peptides like GE11 and its analogues, and nucleic acid aptamers, all of which enhance tumor-specific accumulation and internalization of drug-loaded carriers [63, 64]. In parallel, albumin-based nanoparticles have gained prominence as biocompatible carriers capable of improving drug solubility, circulation time, and tumor penetration. HSA nanoparticles, exemplified by lapatinib-loaded formulations, demonstrate enhanced drug delivery across the blood–brain barrier and superior inhibition of TNBC metastasis by leveraging albumin’s natural affinity for tumor-associated receptors such as gp60 and SPARC [61]. Furthermore, functionalized albumin nanoparticles, such as anacardic acid–modified self-assembled systems co-delivering docetaxel and gemcitabine, show synergistic effects by targeting multiple oncogenic pathways and improving pharmacokinetic profiles [65]. Combining EGFR-targeted delivery with albumin-based nanocarriers thus offers a powerful dual strategy: the targeting ligand directs the carrier to EGFR-overexpressing tumor sites, while albumin ensures stability, biodegradability, and efficient intracellular transport. This integrated design enhances therapeutic efficacy, reduces systemic toxicity, and holds great promise for overcoming resistance and metastasis in aggressive breast cancer subtypes.

#### CD44

Numerous studies have examined the function of CD44 as a biomarker and therapeutic target in TNBC, revealing that aggressive breast cancer phenotypes are associated with protein overexpression. In the context of nanotechnology, several interventions have drawn much interest because of their potential to improve targeted medicine delivery and lower systemic toxicity. Among these, albumin-based nanoparticles have shown encouraging promise for targeted therapy mediated by CD44. Although CD44 is classically recognized as a receptor for hyaluronic acid (HA), recent evidence demonstrates that acetylated forms of human serum albumin (HSA) can also activate CD44 receptors through interactions that mimic the acetyl group–mediated binding mechanism of HA. Xiong et al. showed that acetyl-lysine HSA nanoparticles selectively enter CD44-positive cancer stem cells via receptor-mediated endocytosis, achieving complete tumor eradication with minimal toxicity [66]. This finding provides mechanistic support for the rationale of employing albumin nanocarriers in CD44-targeted delivery.

For instance, Cirillo et al. developed cationized and redox-responsive HSA nanoparticles complexed with HA to facilitate CD44-mediated delivery of doxorubicin (DOX). The resulting nanoparticles (FNPs) were monodisperse, with an average size of approximately 240 nm and a positive zeta potential of + 15.4 mV, ensuring colloidal stability. They achieved a high DOX loading efficiency of about 90 percent and exhibited redox-triggered drug release, with 55 percent released within two hours under glutathione (GSH)-mimicking tumor conditions. Cellular uptake and cytotoxicity were significantly enhanced in TNBC cell lines expressing high CD44 levels, where DOX-loaded FNPs disrupted glycolytic and oxidative phosphorylation pathways, leading to tumor cell death, reduced invasiveness, and inhibition of mammosphere formation [59]. Similarly, Li et al. synthesized cationic bovine serum albumin nanoparticles decorated with HA for targeting CD44-overexpressing cancer stem cells (CSCs). These nanoparticles loaded with all-trans retinoic acid (ATRA) demonstrated ultrahigh drug encapsulation efficiency (~ 93%) and showed selective uptake in CD44-rich tumor cells both in vitro and in vivo. Importantly, HA-mediated targeting improved therapeutic specificity, which translated into reduced tumor volumes and inhibition of CSCs in mouse metastasis models, with minimal off-target toxicity. The HA coating not only facilitated active targeting via CD44 but also improved nanoparticle stability and circulation time, providing an effective platform for CSC-targeted therapy in breast [67].

#### CXCR4

TNBC is characterized by the absence of estrogen, progesterone, and HER2 receptors, rendering hormone therapies and HER2-targeted treatments ineffective. Consequently, chemotherapy remains the mainstay systemic treatment. Due to its aggressive nature and lack of targeted receptors, TNBC cells often exhibit overexpression of alternative targets such as the chemokine receptor CXCR4, which is being actively investigated for targeted therapy development [68].

Albumin-based nanoplatforms that specifically overexpress the CXCR4 receptor can be engineered to target TNBC cells. In this type of delivery system, anticancer drugs are delivered directly to the tumor site, and these nanoparticles are able to increase the effectiveness of the therapy with minimal side effects [69]. Albumin is the best material for making these drug delivery nanoparticles because it is biocompatible, biodegradable, nontoxic, and nonimmunogenic.

One of them includes surface modification of albumin nanoparticles via the use of tumor-homing peptides to selectively target TNBC cells. Nanoparticles have also been engineered to deliver chemotherapeutic drugs, such as doxorubicin, more efficiently than conventional approaches [69]. Nanoparticle-based drug delivery systems have several benefits:

- *Enhanced permeability and retention (EPR) effect*: Nanoparticles can accumulate in tumor tissue because of the leaky vasculature and impaired lymphatic drainage.
- *Targeted delivery*: Functionalizing nanoparticles with ligands that specifically bind to receptors that are overexpressed on cancer cells, such as CXCR4, allows targeted drug delivery [70, 71].
- *Improved bioavailability*: Nanoparticles can protect drugs from degradation and increase their circulation time in the bloodstream.
- *Reduced Toxicity*: Selective targeting of cancer cells can reduce the exposure of healthy tissues to toxic chemotherapeutic agents, minimizing side effects [72].

One study used electrohydrodynamic cojetting to prepare multicompartmental drug delivery carriers for CXCR4 targeting. These particles consist of a poly(lactide-co-glycolide) derivative that has the ability to immobilize Plerixafor. It is a small molecule that has an inhibitory effect on CXCR4 [70].

Albumin-based nanoplatforms may be used for both diagnosis and treatment. For example, albumin-functionalized mesoporous silica nanoparticles may be used to deliver cytotoxic drugs via these imaging modalities, and concurrent treatment of TNBC can be monitored [73].

CXCR4-targeted liposomal preparations may also augment immune checkpoint blockade (ICB) therapy for TNBC. The incorporation of CXCR4 increases immunosuppression activity in the tumor microenvironment, and CXCR4-targeted therapies can reverse this process [71].

Although albumin-based nanoplatforms have the greatest potential for TNBC cell-targeted drug delivery, challenges still exist. Optimization is important, such as improving the size, shape, and surface properties of the nanoparticles, which improve tumor penetration and release of the drug. More research can be conducted on the long-term safety and efficacy of these nanoplatforms in clinical trials [69].

## Drug loading and therapeutic payloads

### Drug loading mechanisms and binding strategies

Albumin-based nanoplatforms utilize multifunctional drug loading methods and binding modes that support efficient, targeted delivery of anticancer drugs in triple-negative breast cancer (TNBC) [74]. Drugs can be loaded onto albumin nanocarriers via two methods: physical encapsulation or covalent conjugation. Physical loading takes advantage of the inherent binding sites and hydrophobic pockets of albumin to noncovalently bind hydrophobic drugs, which enhances their solubility and stability. Covalent conjugation chemically attaches drugs to albumin with the help of cleavable linkers that respond to tumor-specific stimuli such as low pH, hypoxia, or tumor-overexpressing enzymes, which allows targeted drug release in the tumor microenvironment with reduced systemic toxicity. The interaction of albumin with receptors that are overexpressed in TNBC tissues, such as gp60 and SPARC, allows for active targeting and transcytosis of drug-loaded nanoplatforms to tumor locations. This combination of noncovalent and covalent binding, along with stimuli-responsive release mechanisms, highlights the potential of albumin-based nanoplatforms for enhancing the therapeutic index and selective delivery of anticancer drugs in TNBC treatment.

### Albumin-encapsulated liposomes

Albumin-encapsulated liposomes have emerged as a highly promising platform for delivering paclitaxel against breast cancer, combining the enhanced solubility and tumor-targeting features of nab-paclitaxel with the advantages of stability and controlled release of liposomal carriers [21, 75]. Unlike traditional paclitaxel formulations that rely on toxic solvents, these hybrid nanocarriers exploit the enhanced permeability and retention (EPR) effect to passively accumulate in tumor tissue, achieving higher intratumoral drug concentrations and minimizing systemic toxicity [76, 77]. In 2D-cultured MCF-7 cells, paclitaxel-loaded albumin nanoparticle-encapsulated liposomes demonstrated 30–40% greater cytotoxicity (lower IC₅₀ values) than paclitaxel-albumin nanoparticles did, reflecting improved cellular uptake via clathrin-mediated endocytosis and energy-dependent pathways [75]. Similarly, in 3D-cultured MDA-MB-231 spheroids—an aggressive triple-negative breast cancer (TNBC) model lacking ER, PR, and HER2 receptors—albumin-encapsulated liposomes reduced spheroid viability by more than 50% at equivalent drug doses, outperforming conventional Abraxane® by sustaining drug release within the hypoxic core and overcoming multidrug resistance mechanisms. Surface modification with polyethylene glycol (PEG) and tumor-targeting ligands such as folate or RGD peptides can prolong the circulation half-life and enable active targeting to folate receptor-overexpressing tumors, leading to an additional 20% increase in tumor uptake over passive EPR targeting [78], whereas pH-sensitive linkers in the albumin–drug conjugate facilitate acid-triggered release within endosomal compartments, ensuring site-specific drug liberation [79]. Innovative combination strategies—such as coloading doxorubicin and curcumin to achieve up to a fourfold reduction in IC₅₀ in MCF-7 cells and embedding IR-780 for NIR-triggered hyperthermia, which accelerates drug release and increases tumor accumulation by 70% demonstrate the platform’s versatility [80]. In addition to chemotherapeutics, radioisotope-labeled albumin-encapsulated liposomes (e.g., ^131^I-labeled) have been leveraged for internal radiotherapy of 4T1 breast tumors, where a single pretreatment enhances vascular permeability and facilitates twofold greater accumulation of subsequent anti-PD-L1 and hypoxia-activated prodrug (AQ4N) therapies, improving overall survival. Advances also include MRI/NIR-guided photothermal–immunotherapy platforms, PI3Kγ inhibitor + paclitaxel codelivery systems that repolarize tumor-associated macrophages when combined with anti-PD-1, folate-targeted siRNA carriers that achieve 75% VEGF silencing, SPARC-responsive doxorubicin prodrugs with fourfold greater release in SPARC-overexpressing cells, and ultrasound-activated HSA-PEG liposomes that double payload uptake in MCF-7 and MDA-MB-231 cells, collectively underscoring the use of albumin-encapsulated liposomes as a multifunctional, multimodal nanomedicine strategy for breast cancer treatment [78].

### Conventional chemotherapeutics

Chemotherapy remains the primary systemic treatment option, but conventional formulations often suffer from poor solubility, rapid clearance, off-target toxicity, and multidrug resistance. Nanotechnology, particularly albumin-based nanoplatforms, provides an opportunity to overcome these limitations by enhancing solubility, circulation time, and tumor-specific accumulation (Table 1).

**Fig. 4:**
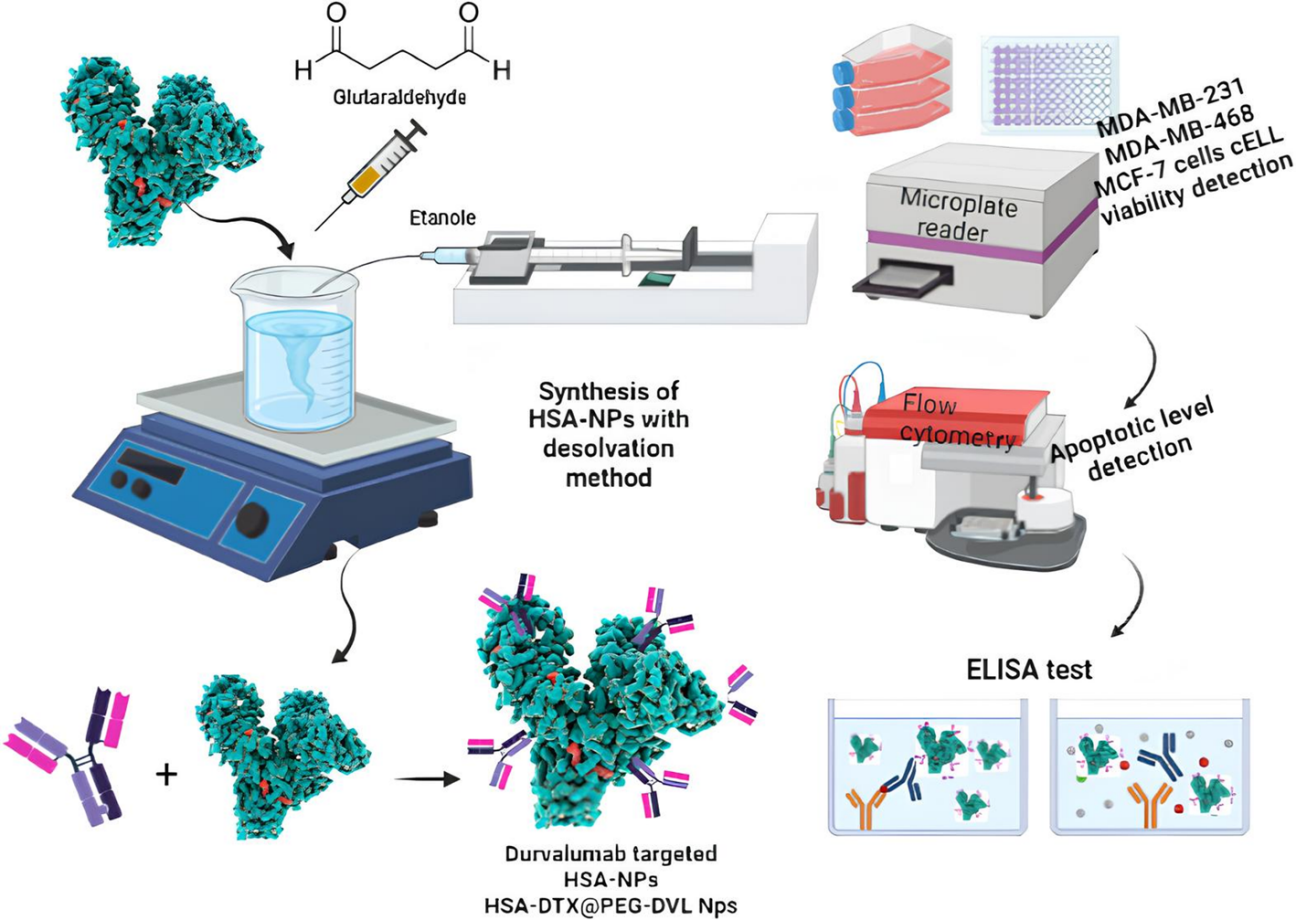
Schematic representation of the synthesis, functionalization, and evaluation of durvalumab-targeted HSA nanoparticles (HSA-NPs). HSA-NPs were prepared via the desolvation method with ethanol and glutaraldehyde as crosslinkers. The nanoparticles were further functionalized with durvalumab (anti-PD-L1 antibody) to generate HSA-DTX@PEG-DVL NPs. Biological assessments included cell viability assays in MDA-MB-231, MDA-MB-468, and MCF-7 breast cancer cell lines via a microplate reader; apoptosis level detection via flow cytometry; and ELISA-based assays for nanoparticle–antibody interactions. Copyright © 2023 The Yurt et al. [81] Published by American Chemical Society

Fatma Yurt and colleagues demonstrated that albumin-based nanoplatforms serve as exceptional drug delivery systems for chemotherapeutic agents targeting triple-negative breast cancer (TNBC) through their innovative development of docetaxel-loaded durvalumab-targeted human serum albumin nanoparticles. Their systematic research revealed that HSA-DTX@PEG-DVL nanoparticles achieved a remarkable drug loading efficiency of 91.8 ± 3.9%, with a loading capacity of 2.2 ± 1%, while maintaining optimal particle sizes between 130 and 178 nm and highly negative zeta potentials (-30 to -32 mV) that ensure colloidal stability. Compared with free docetaxel, the nanoplatform demonstrated superior cytotoxicity across multiple TNBC cell lines (MDA-MB-468, MDA-MB-231, and MCF-7), with IC50 values reduced to 7.5 μg for the targeted nanoformulation versus 15–30 μg for conventional docetaxel. Remarkably, the research team established that their albumin nanocarriers facilitated controlled drug release, achieving 73% release at a physiological pH of 7.4 and 87% release at a tumor-acidic pH of 5.4 over 72 h, enabling selective therapeutic action in the tumor microenvironment while minimizing systemic exposure (Fig. 4) [81].

Hebao Yuan and colleagues pioneered groundbreaking research demonstrating that, compared with conventional taxane formulations, albumin nanoparticle formulations of paclitaxel (Abraxane) fundamentally alter cancer stem cell dynamics in triple-negative breast cancer. Their comprehensive investigations revealed that while Abraxane achieved 3- to fivefold lower blood drug concentrations than Taxol did, it maintained similar tumor drug concentrations with a remarkable tenfold higher tumor-to-plasma ratio, indicating superior tissue penetration and retention capabilities. Most significantly, Yuan’s team established that Abraxane decreased breast cancer stem cell frequency by 3- to ninefold through increased intracellular drug uptake (3- to 15-fold) in both ALDH + cancer stem cells and differentiated ALDH- cells, whereas conventional Taxol paradoxically increased cancer stem cell populations. Mechanistic studies have demonstrated that an albumin-mediated nanoparticle formulation, rather than simple albumin-drug mixtures, is critical for achieving enhanced cellular uptake, providing compelling evidence that the nanoformulation process itself has therapeutic superiority in eliminating metastasis-driving cancer stem cells [82].

Nima Beheshtizadeh and research collaborators comprehensively analyzed the transformative potential of docetaxel-loaded nanoplatforms in oncology, establishing that albumin-based delivery systems represent a paradigm shift in chemotherapeutic efficacy for cancer treatment. Their extensive review highlighted that docetaxel nanoplatforms overcome critical limitations of conventional chemotherapy, including poor solubility (0.025 μg/ml), rapid elimination, and nonspecific toxicity, through sophisticated encapsulation methods that enable targeted delivery with controlled release profiles. The research team emphasized that albumin nanocarriers provide exceptional biocompatibility, reproducible synthesis, and adjustable size characteristics during production while facilitating passive tumor targeting through enhanced permeation and retention (EPR) effects and active targeting via surface modifications with specific ligands. Their analysis revealed that docetaxel-loaded albumin nanoplatforms achieve superior antitumor efficacy by selectively delivering therapeutic payloads to target tissues, reducing systemic side effects and drug dosage requirements while extending the circulation half-life and improving bioavailability at tumor sites [83].

### Natural products and phytochemicals

Nanotechnology-based delivery of phytochemicals such as curcumin, resveratrol, and 3,4-difluorobenzylidene curcumin (CDF) via albumin nanoparticles has demonstrated significant promise in overcoming the inherent limitations of the poor water solubility, rapid metabolism, and nonspecific biodistribution of these natural products in TNBC models. For example, BSA-CDF-ATZ nanoparticles, synthesized by conjugating bovine serum albumin (BSA) with the carbonic anhydrase IX inhibitor acetazolamide (ATZ), achieved particle sizes of 140–180 nm and selectively accumulated in hypoxic TNBC regions through CA-IX targeting. Under hypoxic conditions, these particles triggered pronounced apoptosis in MDA-MB-231 cells, reducing cell viability by more than 70% at 10 µM CDF equivalent, whereas free CDF showed only a 30% reduction under the same conditions [84].

Similarly, curcumin-loaded HSA nanoparticles functionalized with a PD-L1–binding peptide were engineered to specifically target PD-L1–overexpressing TNBC cells. These formulations measured 197 ± 5 nm before peptide conjugation and 246 ± 7 nm postconjugation, with zeta potentials shifting from –25 mV to –18 mV, indicating successful surface modification. In vitro assays revealed a twofold increase in the cellular uptake of PD-L1–high MDA-MB-468 cells compared with that of free curcumin and a corresponding reduction in the IC₅₀ from 12 µM to 5 µM, highlighting the efficacy of active targeting in enhancing cytotoxicity [85].

Moreover, zein–BSA core–shell nanoparticles coencapsulated curcumin and resveratrol through a pH-driven assembly method achieved high drug loading efficiencies (85% for curcumin and 60% for resveratrol) and sustained sequential release: an initial 40% release of resveratrol at pH 7.4 over 24 h, followed by 65% curcumin release at pH 5.5 over 72 h. This design not only stabilized both phytochemicals against premature degradation but also mimicked combination therapy by temporally staggering their release profiles. Cytotoxicity studies in MDA-MB-231 spheroids demonstrated a 50% greater reduction in spheroid volume with the coencapsulated formulation than with equimolar free combinations, underscoring the therapeutic advantage of codelivery within a single albumin-based nanocarrier [86].

In addition to these foundational approaches, Fengjie Liu and colleagues developed baicalin-loaded bifunctional albumin nanoparticles (BANPs) to increase the delivery of the natural flavonoid baicalin for the treatment of triple-negative breast cancer (TNBC). They engineered a core–shell nanostructure of approximately 79.6 ± 1.9 nm with a zeta potential of –33.7 ± 0.8 mV, achieving an encapsulation efficiency of 72.7% and demonstrating acid-sensitive release that accelerated payload discharge in the tumor microenvironment. In vitro studies on MDA-MB-231 cells revealed that, compared with free baicalin, BANPs significantly increased cellular uptake, induced G₀/G₁ cell cycle arrest and early apoptosis, and markedly inhibited proliferation. In 4T1 tumor-bearing mice, systemic administration of BANPs led to prolonged circulation, enhanced tumor accumulation, and superior tumor growth suppression relative to free baicalin. Moreover, BANPs remodeled the inflammatory microenvironment by reducing regulatory T-cell infiltration, promoting the recruitment of macrophages with an anti-inflammatory M2 phenotype, and augmenting M2 macrophage phagocytic activity, collectively contributing to robust antitumor immunity. These findings highlight the potential of albumin-based nanocarriers to improve the bioavailability, target release, and immunomodulatory effects of phytochemicals in TNBC therapy [87].

Together, these albumin nanoparticle platforms illustrate how tailored surface modifications (e.g., antibody or peptide ligands), hypoxia-responsive targeting, and controlled sequential release enable phytochemicals to overcome pharmacokinetic barriers, selectively accumulate in TNBC tumors, and exert enhanced antitumor activity while minimizing off-target effects.

### Molecular and genetic therapeutics

Molecular and genetic therapeutics delivered via albumin nanoparticles represent a cutting-edge approach for triple-negative breast cancer (TNBC), aiming to address the aggressive nature and therapeutic resistance of this subtype through precise gene modulation. Among the forefront of these innovations, small interfering RNA (siRNA) therapies harness the RNA interference pathway to silence oncogenes critical to TNBC progression. Recent studies have demonstrated that lipid-conjugated siRNAs engineered for optimal in situ binding to serum albumin exhibit dramatically enhanced pharmacokinetics and tumor accumulation, with up to 12-fold increased delivery to orthotopic TNBC tumors compared with unconjugated siRNAs. This strategy has enabled approximately 80% silencing of antiapoptotic genes such as MCL1, resulting in significant tumor growth inhibition and improved survival in preclinical TNBC models [88].

Delivery systems leveraging human serum albumin nanoparticles (HSA-NPs) have shown promise as nonviral vectors for siRNA, providing protection from enzymatic degradation and efficient cellular uptake. Compared with free siRNA, HSA-coated lipid nanoparticles loaded with siRNA demonstrated significant gene knockdown in various breast cancer cell lines, including aggressive TNBC variants, and achieved tumor-specific gene silencing in xenograft models with superior pharmacokinetics. Advancements in nanoplatform design incorporate sophisticated targeting ligands to increase tumor specificity, lysosomal escape modalities to improve cytoplasmic delivery, and stimuli-responsive release mechanisms to maximize therapeutic gene silencing within tumor cells [89, 90].

In addition to the use of siRNAs, the use of CRISPR/Cas9 genome editing technology encapsulated in albumin-based nanoparticles has emerged as a potent approach to directly correct or disrupt genetic drivers of TNBC. CRISPR-nano complexes facilitate high-contrast imaging and efficient tumor targeting, overcoming classical delivery challenges related to biodegradability and off-target effects. Preclinical investigations highlight CRISPR-mediated knockouts of resistance genes and epigenetic regulators in TNBC, curbing tumor growth and metastasis. The integration of artificial intelligence and machine learning methods further refines CRISPR design, enhancing precision and therapeutic outcomes [91].

Additionally, albumin nanoparticles serve as codelivery platforms for genetic agents combined with conventional chemotherapeutics. Multifunctional nanoparticles coencapsulating siRNAs and drugs such as paclitaxel or PI3Kγ inhibitors achieve synergistic effects by simultaneously modulating tumor microenvironment immunosuppression and cancer cell survival pathways. These combination approaches repolarize immunosuppressive macrophages, enhance cytotoxic T-cell activation, and induce long-term tumor remission in aggressive breast cancer models [92].

Overall, albumin-based nanocarriers offer biocompatible, scalable, and tumor-targeted solutions for delivering molecular and genetic therapeutics in TNBC. Owing to their ability to protect and selectively transport nucleic acids and genome editing tools, combined with controlled release and immune modulation, these materials are highly promising candidates for overcoming therapeutic resistance, reducing systemic toxicity, and extending patient survival in this challenging cancer subtype.

## Preclinical and clinical evidence

Nab-paclitaxel, an albumin-bound nanoparticle formulation of paclitaxel, has emerged as a critical component in the treatment of triple-negative breast cancer (TNBC), particularly in combination with immune checkpoint inhibitors such as atezolizumab. Recent clinical trials and real-world studies have evaluated its efficacy and safety, highlighting both its potential and challenges. The combination of atezolizumab and nab-paclitaxel has been extensively studied in metastatic TNBC (mTNBC). A meta-analysis of six randomized controlled trials (RCTs) demonstrated that this combination significantly improved progression-free survival (PFS) (HR 0.72, 95% CI 0.59–0.87, p = 0.0006) and the objective response rate (ORR) (RR 1.25, p < 0.00001) compared with nab-paclitaxel alone [93]. However, the overall survival (OS) benefit was not statistically significant (HR 0.90, p = 0.08), suggesting that while the combination delays disease progression, its impact on long-term survival requires further investigation. The safety profile of this regimen revealed increased immune-related adverse events (AEs), including dermatological, pulmonary, endocrine, and neurological toxicities, although hematological and gastrointestinal AEs were comparable to those of nab-paclitaxel monotherapy.

Real-world evidence supports these findings. Fabi et al. (2023), who evaluated atezolizumab plus nab-paclitaxel in PD-L1-positive mTNBC patients, reported a median PFS of 6.3 months and an ORR of 42.3%, which is consistent with the phase III IMpassion130 trial [94]. However, this study also highlighted notable immune-related AEs, such as rash (23.1%), hepatitis (11.5%), and thyroiditis (11.5%), emphasizing the need for careful monitoring in clinical practice. Importantly, the FDA has issued alerts regarding the specificity of the combination, noting that atezolizumab is approved only with nab-paclitaxel (not solvent-based paclitaxel) for PD-L1-positive mTNBC, as efficacy was not demonstrated with paclitaxel in the IMpassion131 trial [94].

In addition to immunotherapy combinations, nab-paclitaxel has been studied with glucocorticoid receptor (GR) antagonists such as mifepristone and relacorilant to overcome chemoresistance. A randomized phase II trial revealed that adding mifepristone to nab-paclitaxel did not improve PFS or ORR in advanced TNBC but was associated with increased grade 3 neutropenia (46% vs. 7%). In contrast, relacorilant (a selective GR modulator) combined with nab-paclitaxel improved PFS and duration of response in patients with platinum-resistant ovarian cancer, suggesting potential for further exploration in TNBC [95].

Elderly patients with TNBC represent a vulnerable population that is often underrepresented in trials. The Ricciardi et al. study (2025) focused on patients ≥ 65 years with HER2-negative mTNBC and reported that nab-paclitaxel monotherapy was effective and well tolerated, with a median PFS of 6 months and a median OS of 40.5 months [96]. Similarly, the Schneeweiss et al. study (2025) confirmed the manageable safety profile of nab-paclitaxel in advanced breast cancer, with high-grade AEs occurring in < 8% of patients [97].

Ongoing challenges include identifying biomarkers for patient selection. While PD-L1 expression is currently used to guide immunotherapy combinations, the ANASTASE study revealed that most responders (97.6%) were PD-L1-negative, suggesting the need for broader biomarkers. Additionally, the Gioan et al. trial (2025) explored a triple combination of atezolizumab, paclitaxel, and bevacizumab in patients with TNBC and reported a median PFS of 11.0 months and OS of 27.4 months, regardless of PD-L1 status. These findings indicate that antiangiogenic agents may synergize with immunotherapy and chemotherapy, although this study used solvent-based paclitaxel, not nab-paclitaxel [98].

When these data are integrated into practice, clinicians must balance the demonstrated PFS and subgroup OS advantages reported in IMpassion130 and corroborated them in subsequent real-world series, with a higher frequency of immune-related high-grade events observed across trials. Table 4 summarizes the key trials discussed here, including trial identifiers, populations, regimens, principal efficacy endpoints, and major safety signals, and can be used as a quick reference to compare absolute outcomes and toxicity patterns across studies (see Table 4). Taken together, these findings support the use of nab-paclitaxel as an effective chemotherapy backbone for PD-L1-directed immunotherapy in patients with metastatic TNBC and emphasize the importance of PD-L1 biomarker assessment, proactive management of immune-mediated toxicity, and continued investigation into optimal sequencing and combinations to maximize benefit with acceptable safety.

**Table 4:** Summary of key clinical trials evaluating immune checkpoint inhibitors and targeted therapies for triple-negative breast cancer (TNBC), including studies on study design, treatment regimens, efficacy outcomes, and safety profiles

ClinicalTrials.gov ID	Phase	Population	Regimen	Key efficacy outcomes	Key safety outcomes
NCT02425891 (IMpassion130)	III	Untreated metastatic/locally advanced TNBC	Atezolizumab 840 mg + nab-paclitaxel 100 mg/m^2^ vs placebo + nab-paclitaxel	PFS (ITT): 7.2 mo vs 5.5 mo; PFS (PD-L1 +): 7.5 mo vs 5.0 mo; OS (PD-L1 +): 25.0 mo vs 15.5 mo	↑ serious AEs, high-grade AEs, dermatologic, pulmonary, endocrine, neurologic AEs with atezo + nab-paclitaxel; no difference in hematologic or GI AEs
NCT04148911 (EL1SSAR)	IIIb	PD-L1 + unresectable locally advanced/metastatic TNBC	Atezolizumab 840 mg + nab-paclitaxel 100 mg/m^2^ (single-arm)	ORR: 42.3% (95% CI 28.9–55.7%); real-world median PFS: 6.3 mo	Grade ≥ 3 AEs; Grade ≥ 2 immune-mediated AEs; long-term safety follow-up to 4.5 yr
NCT02530489	II	Neoadjuvant TNBC nonresponders to anthracycline	Atezolizumab + nab-paclitaxel (neoadjuvant → surgery → atezo adjuvant)	pCR/RCB-0 + RCB-I rate; pCR 38.5%	Adverse events: manageable; specific rates not reported
NCT03121352	I/II	Metastatic TNBC	Carboplatin AUC 4.5 + nab-paclitaxel 75 mg/m^2^ + pembrolizumab 200 mg	ORR: 68.1% (CR 27.4%, PR 40.7%); DCR: 77.8%; mean PFS: 5.8 mo	SAEs: 43.3%; most common: pneumonia 6.7%, dyspnea 10.0%, immune-related hepatitis 3.3%
NCT04251533 (EPIK-B3)	–	Advanced TNBC with PIK3CA mutation/PTEN loss	Alpelisib 300 mg + nab-paclitaxel 100 mg/m^2^ vs placebo + nab-paclitaxel	PFS: 7.2 mo vs 5.6 mo; ORR: 40.4% vs 34.0%; CBR: 50.0% vs 44.0%	All-cause mortality ~ 12%; SAEs: 42.3% vs 30.0%; frequent high-grade events in experimental arm

## Challenges and limitations

Albumin-based nanoplatforms hold immense promise for targeted drug delivery in triple-negative breast cancer (TNBC); however, their clinical translation is hampered by several critical challenges that must be addressed for improved therapeutic efficacy. One of the foremost concerns lies in drug loading and delivery efficiency, as the limited payload capacity of albumin nanoparticles often restricts the amount of therapeutic agent that can be carried, potentially necessitating higher or more frequent doses, thereby increasing the risk of systemic toxicity [72, 99]. This is highly related to the problem of release kinetics, as the controlled and site-specific release of drugs is central to optimizing therapeutic effects and reducing toxicity. In addition to delivery efficacy, the structural integrity of albumin nanocarriers under dynamic physiological conditions represents another considerable challenge. These include pH, ionic strength, and enzymatic variation, which can disrupt the integrity of the nanoparticle, induce aggregation or premature degradation, and emphasize the need for maximized surface modification to extend the circulation time. Concurrently, high tumor specificity continues to be elusive, with the reliance on the enhanced permeability and retention (EPR) effect being too often inadequate owing to interpatient heterogeneity in the tumor vasculature [100]. Whereas active targeting approaches, such as ligand or antibody functionalization, have been promising, the selection and display of these moieties are still delicate undertakings. In addition, whereas albumin is inherently biocompatible, chemical alterations can impart immunogenicity risks, thereby requiring extensive scrutiny of immune reactions and long-term safety [101]. These difficulties increase the heterogeneity of TNBC itself. The heterogeneous molecular subtypes make the use of a one-size-fits-all albumin-based platform unviable, with subtype-specific nanocarrier designs necessitating deeper subtype-specific molecular knowledge. The physiological heterogeneity between patients, for example, variability in renal and hepatic functions and renal functions, impacts the metabolism and clearance of albumin-drug conjugates and affects bioavailability [102]. Last, their translation from bench-scale innovation to universal clinical application on the basis of the creation of scale-up, affordable manufacturing techniques, as dependence on expensive materials might compromise availability or as intricate synthesis protocols. Cumulatively, these challenges are multifaced and highlight the need for a concerted strategy that integrates rational design, tumor biology knowledge, and sophisticated manufacturing to comprehensively unlock the therapeutic potential of albumin-based nanomedicines in TNBC. These challenges are overcome by considering TNBC’s very nature as a heterogeneous disease, with disparate molecular subtypes invalidating the notion of a one-size-fits-all albumin-based platform, which requires a subtype-specific nanocarrier design on the basis of a more profound molecular understanding. The physiological differences between patients, which include renal and hepatic functions, affect the elimination of albumin‒drug conjugates and metabolism, thus affecting overall bioavailability.

**Fig. 5:**
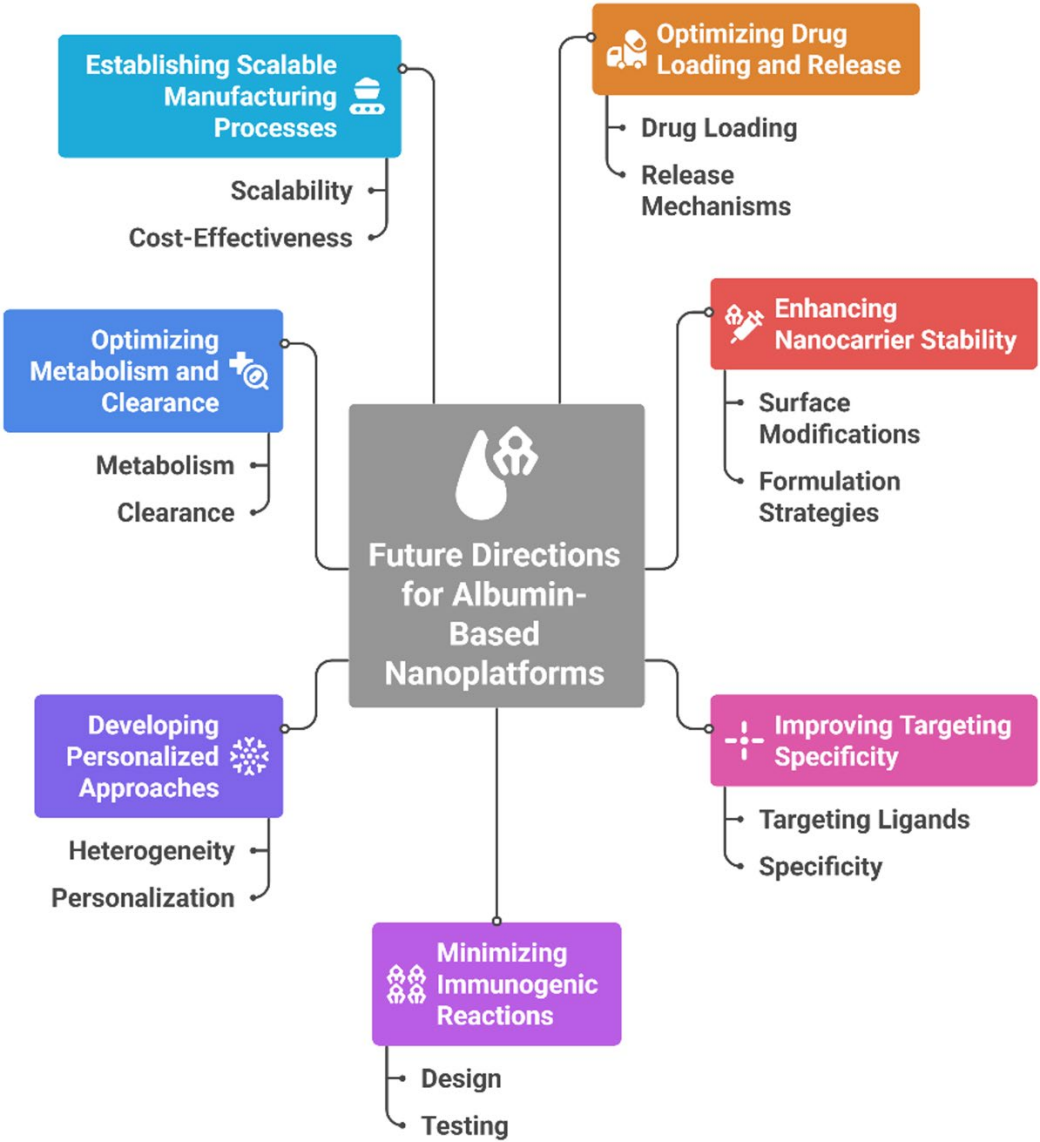
Key future directions for albumin-based nanoplatforms, illustrating solutions such as improving drug loading, stability, targeting specificity, personalization, metabolism, immunogenicity, and scalable manufacturing

Figure 5 summarizes the key future directions and potential solutions for overcoming the challenges faced by albumin-based nanoplatforms in targeted drug delivery for triple-negative breast cancer (TNBC). This review highlights essential strategies, including optimizing drug loading and release mechanisms, enhancing nanocarrier stability, improving targeting specificity, minimizing immunogenic reactions, developing personalized approaches to address TNBC heterogeneity, optimizing metabolism and clearance, and establishing scalable, cost-effective manufacturing processes.

## Conclusion

The development of albumin-based nanoplatforms offers a transformative avenue for targeted therapy in TNBC, marked by significant advancements and ongoing challenges. Future directions emphasize personalized nanomedicine approaches tailored to address the molecular heterogeneity of TNBC, thereby enabling more precise and effective treatments. The design of multistimuli-responsive albumin nanocarriers that react to specific tumor microenvironment cues could enhance drug release control and therapeutic outcomes. The integration of albumin nanoplatforms with cutting-edge modalities such as gene editing and immunotherapy may increase anticancer efficacy through synergistic mechanisms. Furthermore, the incorporation of artificial intelligence and multiomics data analysis in nanoplatform design will facilitate the rational optimization of these systems for individual patient profiles, supporting precision oncology and real-time treatment monitoring. In conclusion, albumin-based nanocarriers combine biocompatibility, functional versatility, and intrinsic tumor-targeting features to address key limitations in conventional TNBC therapy. These nanoplatforms improve drug delivery specificity, circumvent resistance mechanisms, and stabilize sensitive therapeutic agents. Nonetheless, challenges such as drug loading limitations, stability under physiological conditions, targeting specificity, immune interactions, and tumor heterogeneity remain critical barriers. Balancing these challenges with multifaceted opportunities requires integrated research efforts aimed at novel formulations, personalized strategies, and scalable production. Continued innovations in albumin nanomedicine hold substantial promise for advancing clinical translation and improving outcomes in TNBC patients.

## Data Availability

No datasets were generated or analyzed during the current study.

## Abbreviations

ALDH: Aldehyde Dehydrogenase
ATRA: All-trans Retinoic Acid
CARP-1: Cell Cycle and Apoptosis Regulatory Protein 1
CDF: 3,4-Difluorobenzylidene Curcumin
CD44: Cluster of Differentiation 44
CSCs: Cancer Stem Cells
CTLA-4: Cytotoxic T-Lymphocyte-Associated Protein 4
DCR: Disease Control Rate
DOX: Doxorubicin
ECM: Extracellular Matrix
EDC: 1-Ethyl-3-(3-dimethylaminopropyl) Carbodiimide
EGFR: Epidermal Growth Factor Receptor
EPR: Enhanced Permeability and Retention
FNPs: Functionalized Nanoparticles
FcRn: Neonatal Fc Receptor
GSH: Glutathione
HA: Hyaluronic Acid
HSA: Human Serum Albumin
ICB: Immune Checkpoint Blockade
IC50: Half Maximal Inhibitory Concentration
ICIs: Immune Checkpoint Inhibitors
IR: Infrared
kDa: Kilodalton
LAR: Luminal Androgen Receptor
MAPK: Mitogen-Activated Protein Kinase
MDR: Multidrug Resistance
MCF-7: Michigan Cancer Foundation-7 (breast cancer cell line)MDA-MB-231
